# Understanding African American/Black and Latine young and emerging adults living with HIV: a sequential explanatory mixed methods study focused on self-regulatory resources

**DOI:** 10.1186/s12939-025-02492-5

**Published:** 2025-05-05

**Authors:** Leo Wilton, Marya Gwadz, Charles M. Cleland, Stephanie Campos, Michelle R. Munson, Caroline Dorsen, Samantha Serrano, Dawa Sherpa, Shaddy K. Saba, Corey Rosmarin-DeStefano, Prema Filippone

**Affiliations:** 1 Department of Human Development, State University of New York at Binghamton, 4400 Vestal Parkway East, Binghamton, NY 13902 USA; 2 Faculty of Humanities, University of Johannesburg, PO Box 524, Auckland ParkJohannesburg, 2006 South Africa; 3 Silver School of Social Work, New York University, 1 Washington Square North, New York, NY 10003 USA; 4 Department of Population Health, New York University Grossman School of Medicine, NYU Langone Health, 180 Madison Avenue, 2-53, New York, NY 10016 USA; 5 85 Lexington Ave, Jersey City, NJ 70304 USA; 6 NYU Rory Meyers College of Nursing, 433 1 Avenue, New York, NY 10010 USA; 7 North Jersey Community Research Initiative, 393 Central Avenue, Newark, NJ 07103 USA

**Keywords:** HIV care continuum, HIV viral non-suppression, Mixed methods, Qualitative, Young adult, Emerging adult, Black, Latino/Latine, Substance use, Harm reduction, Mental health, Social action theory

## Abstract

**Background:**

HIV care continuum engagement is inadequate among African American/Black and Latine (AABL) young/emerging adults living with HIV in the United States. Within this population, some subgroups face barriers to research and are under-studied. Grounded in social action theory, the present study focuses on a diverse community-recruited cohort including those with non-suppressed HIV viral load. Using a sequential explanatory mixed methods design, we describe contextual self-regulatory resources (e.g., substance use, mental health), and their relationships to HIV management.

**Methods:**

Participants (*N* = 271) engaged in structured baseline assessments and biomarker testing (HIV viral load, drug screening). Being well-engaged in HIV care and HIV viral suppression were the primary outcomes. We purposively sampled a subset for maximum variability for in-depth interviews (*N* = 41). Quantitative data were analyzed via descriptive statistics and logistic regression, and results were used to develop qualitative research questions. Then, qualitative data were analyzed via directed content analysis. The joint display method was used to integrate results.

**Results:**

Participants’ mean age was 25 years (SD = 2). The majority (59%) were Latine/Hispanic and 41% were African American/Black. Nearly all were assigned male sex at birth (96%) and identified as gay/bisexual/queer (93%). The average HIV diagnosis was 4 years prior (SD = 3). The majority were well-engaged in HIV care (72%) and evidenced viral suppression (81%). Substance use (tobacco, marijuana, alcohol) was prevalent, mainly at low- and moderate-risk levels. Drug screening indicated marijuana, methamphetamine, and MDMA were the most common recent substances. Symptoms of depression and PTSD were associated with decreased odds of engagement in care. High-risk cannabis use was associated with decreased odds of HIV viral suppression. Qualitative results highlighted the prevalence of substance use in social networks and venues, and the importance of substances as a coping strategy, including for mental health distress. Tobacco and methamphetamine (but not marijuana) were described as problematic, and marijuana was used as harm reduction. Substance use was more common among those with non-suppressed versus suppressed HIV viral load. However, overall, substance use did not commonly interfere substantially with HIV management.

**Conclusions:**

The present study advances knowledge on AABL young/emerging adults living with HIV and highlights ways to improve screening and services.

**Supplementary Information:**

The online version contains supplementary material available at 10.1186/s12939-025-02492-5.

## Introduction

There is a consensus that to end the HIV epidemic in the United States, 95% of people living with HIV need to be diagnosed, 95% of diagnosed individuals need to take HIV antiretroviral therapy, and 95% of those on HIV antiretroviral therapy need to evidence HIV viral suppression [1, 2]. Further, sustaining HIV viral suppression consistently over time is necessary for wellbeing, longevity, and to prevent transmission of HIV to others [1, 2]. However, over the course of the HIV epidemic, disparities in engagement along this HIV care continuum related to race/ethnicity and age have been serious and persistent [3, 4]. Compared to their White counterparts, African American/Black and Latine (AABL) young and emerging adults living with HIV are less likely to engage in HIV care and take HIV medication with high levels of adherence [3, 4]. Further, AABL young and emerging adults evidence lower rates of sustained HIV viral suppression than their White counterparts [5]. For example, in a national study, rates of sustained HIV viral suppression were 36% among African American/Black, 47% among Latine/Hispanic, and 51% among White young and emerging adults [5]. Research on AABL young and emerging adults living with HIV is needed to eliminate these disparities.

The majority of studies with AABL young and emerging adults living with HIV are conducted in medical or clinical settings [6–8]. However, this population is diverse and includes those who are poorly engaged along the HIV care continuum, which reduces their chances of being engaged in research, along with those who experience other barriers to research participation such as fear of HIV status disclosure, substantial medical distrust, concerns about immigration status, language barriers, and stigma associated with non-suppressed HIV viral load [9, 10]. The present study took the approach of recruiting in the community rather than medical settings to enroll a diverse sample of AABL young and emerging adults living with HIV [11, 12], to thereby complement studies conducted in medical settings and advance the literature on this population. In particular we were interested in those with non-suppressed HIV viral load, because of the importance of HIV viral suppression for individual and public health, as noted above, and secondarily, in other under-studied subpopulations including those whose primary language is Spanish, immigrant, refugee, and asylum-seeking persons, and persons with serious socioeconomic disadvantage [9, 10].

**Fig. 1:**
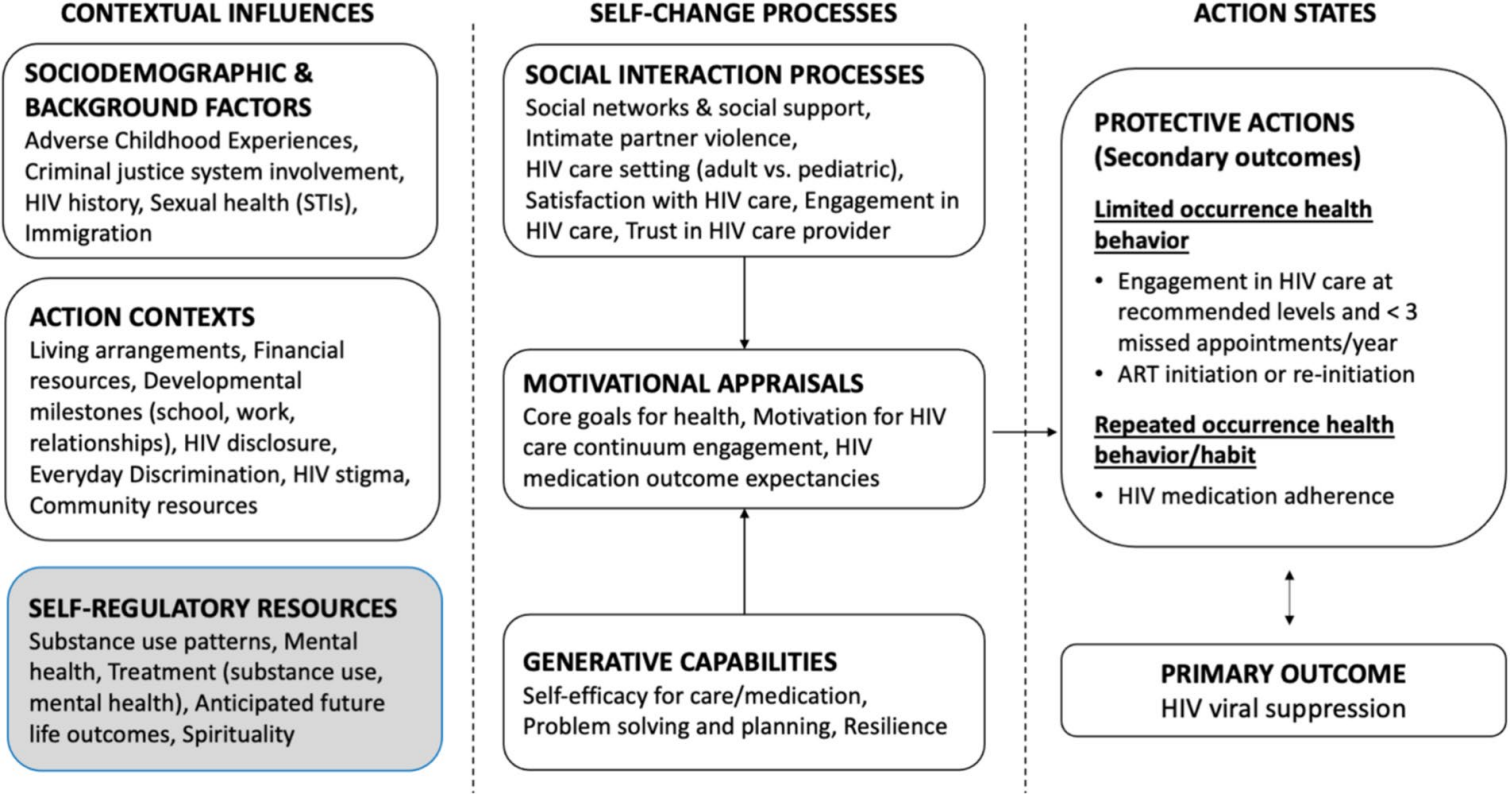
Social action theory model with the domains examined in the present study highlighted

The present study is grounded in social action theory, a comprehensive social-cognitive theory [13]. Social action theory captures how “upstream” contextual influences (called background factors, action contexts, and self-regulatory resources), influence self-change processes at the social and individual levels, which then in turn, affect action states (protective actions) that produce health outcomes, such as HIV viral suppression. These contextual influences may have direct effects on health protective actions and may also influence domains downstream in the model. Social action theory has been adapted in past research with persons living with HIV and young men who have sex with men [14, 15]. Our research team modified social action theory for the population of AABL young and emerging adults living with HIV in order to study HIV management in this population (see Fig. 1). To do so, we took into consideration that young and emerging adulthood are developmental periods of transition and transformation in peer and family relationships, finances, independence, and autonomous management of health and health care [16–18]. Further, we considered that AABL young and emerging adults living with HIV experience stressors and challenges typical of those at these developmental periods, along with atypical difficulties including ongoing HIV management. The social action theory model guiding the present study reflects these considerations.

In a previous paper, we explored two aspects of these contextual influences, namely, background factors and action contexts including discrimination, immigration, adverse childhood experiences, and housing status [9]. The present study explores another set of contextual influences on HIV management, namely, self-regulatory resources. Self-regulatory resources include psychosocial factors, behaviors, and external assets that shape social and behavioral processes found downstream in the conceptual model. The specific self-regulatory resources explored in this paper include substance use behavior, mental health functioning, treatment for substance use and mental health, anticipated future life outcomes, and spirituality. In Fig. 1, we highlight the domains studied in the present paper. The present study focuses in particular on substance use, which is prevalent among young and emerging adults generally and in this population [19, 20], and is of interest because the types and patterns of substances used and their effects on health, as well as a population’s perspectives on substance use, change over time [21, 22]. We also highlight mental health distress, which commonly co-occurs with, and can be a cause or a consequence of substance use [23, 24]. We review the literature on these domains in brief below.

Past research has indicated that AABL young and emerging adults living with HIV evidence higher prevalence rates of substance use and mental health symptoms compared to their peers not living with HIV [25–27]. Marijuana is very common in this population (≥ 50% use it), mainly at non-hazardous levels, but a proportion use heavily [23, 28], along with alcohol [29]. Tobacco use is also highly prevalent, including daily or near daily use (~ 25–48%) [29, 30]. Less common but still widespread are “club drugs” such as MDMA (Ecstasy), GHB, Rohypnol, and ketamine, including in social settings (~ 25%) [29], along with stimulants such as powder and crack cocaine and crystal methamphetamine [29, 31]. Regarding mental health symptoms, depression and anxiety are most common (25–60%) [32], followed by Post-Traumatic Stress Disorder (PTSD) [33]. Both substance use behavior and mental health functioning have the potential to promote or disrupt other domains on the pathway, including HIV management [19, 20]. Access to resources such as substance use and mental health treatment are critical to support downstream self-change processes and action states [18]. Yet, AABL young and emerging adults living with HIV commonly experience barriers to such treatment [34, 35]. Anticipated future life outcomes are a person's subjective beliefs about their future [36]. Past research has shown that anticipated future life outcomes play a role in shaping health behavior [36, 37]. Spirituality is a protective factor among young and emerging adults [38, 39] [40].

The present study focuses on a diverse cohort of AABL young and emerging adults living with HIV recruited in the community, including subgroups that are generally under-studied in research. It takes a mixed methods approach to describe a set of self-regulatory resources and their relationships to HIV management, and to uncover participants’ views on their causes, meanings, and effects.

## Methods

The present study uses baseline quantitative and qualitative data from a larger longitudinal study focused on AABL young and emerging adults living with HIV, including those with and without HIV viral suppression, aged 19–28 years. The larger study was conducted in New York City and Newark, New Jersey (NJ). Participants were enrolled between December 2021 and October 2023 (*N* = 271 individuals, 19% with non-suppressed HIV viral load). A subset was selected for semi-structured qualitative interviews (*N* = 41). Activities were carried out in English and Spanish. The study was conducted by an academic institution in New York City in partnership with the North Jersey Community Research Initiative, a large multi-service community-based organization in Newark, NJ. In-person study activities took place mainly at a field site in lower Manhattan but also in Newark, NJ. Participants were compensated for study activities. The study was approved by the Institutional Review Board at New York University and participants gave informed consent for the study. Methods are detailed elsewhere and reviewed briefly below [9, 10].

### Design

The present study is descriptive and uses a sequential explanatory mixed methods design [41]. This design proceeds in two phases: first, quantitative data are analyzed, followed by qualitative data to contextualize and extend the quantitative findings [41]. We first explored self-regulatory resources using quantitative data, describing those with and without HIV viral suppression, and the relationships between self-regulatory resources to two outcomes: being well-engaged in HIV care and HIV viral suppression. Next, the quantitative findings were used to generate qualitative research questions. In a final step, we integrated the quantitative and qualitative data.

### Recruitment

Participants were recruited with a hybrid strategy that included peer-to-peer recruitment, social media, dating apps, ads placed in public transportation venues, and recruitment in community-based organizations in New York City and Newark, NJ [10].

### Participants

Eligibility criteria were: age 16–28 years; AABL race/ethnicity; residence in the New York City or Newark, NJ metropolitan areas; HIV diagnosis (confirmed with medical documentation); diagnosed with HIV ≥ 3 months ago; HIV was transmitted behaviorally, not perinatally; able to conduct activities in English or Spanish; willing to use their own phone for study activities or a phone provided by the project; and willing to provide assessment of HIV viral load at screening.

### Procedures

#### Screening for eligibility and baseline assessment

Participants provided verbal informed consent and then were screened for eligibility, including HIV viral load testing from a commercial laboratory or by recent lab report from their primary care setting. Among those found eligible, signed informed consent was obtained. Then, the baseline assessment battery (taking 60–90 min) and drug screen by urinalysis were carried out ($60 compensation).

#### Qualitative in-depth interview procedures

To select participants from the larger sample, we took the approach of purposive sampling for maximum variability on key indices including race/ethnicity, language (English vs. Spanish), and HIV viral load status (suppressed vs. non-suppressed). The interview was conducted by phone and in-person between six and twelve months post-enrollment. The interview lasted between 60–90 min. Interviews were conducted by four Master’s and PhD-level qualitative researchers trained in anthropology or public health; three were fully bilingual in English and Spanish. A total of 41 interviews were conducted, with nearly one-third (32%, 13/41) conducted in Spanish. A semi-structured template was used to guide the qualitative interviews, which were audio-recorded and professionally transcribed. Spanish transcriptions were translated into English by a professional service, and then transcripts were checked for accuracy by members of the research team who were fluent in Spanish.

## Measures

### Sociodemographic characteristics and background factors

Demographic characteristics included age (in years, assessed as a continuous variable), gender identity (e.g., man, woman, gender non-binary, transgender, gender fluid, recoded as cisgender vs. transgender, gender expansive, gender non-binary, gender queer or otherwise not cisgender), sexual orientation (e.g., gay, lesbian, straight/heterosexual, pansexual, queer, recoded as lesbian, gay, bisexual, queer, or other non-heterosexual sexual orientation, yes/no), race/ethnicity, and sex assigned at birth (male, female, intersex, other), primary language (English or Spanish), immigration status (recoded as born outside US or Puerto Rico, yes/no) education (coded as less than high school vs. high school graduate or higher), and HIV history; namely, years since HIV diagnosis as a continuous variable [42, 43]. Childhood adversity was measured using a revised version of the Adverse Childhood Experiences (ACES) inventory called the ACES-R [44]. ACES items were coded on a yes/no scale and the sum of affirmative responses was calculated, which comprised the ACES-R score ranging from 0–14 (Cronbach’s α = 0.89). More detail on the demographic characteristics of the sample and how they were assessed is provided elsewhere [9].

### Self-regulatory resources

*Substance use patterns and risk scores.* Substance use patterns were assessed with the World Health Organization Alcohol, Smoking and Substance Involvement Screening Test (WHO ASSIST) [45]. The ASSIST is designed to identify substance use patterns that increase the risk of adverse health outcomes. The ASSIST asks about 11 substances—tobacco products, alcohol, cannabis, cocaine, prescription stimulants, methamphetamine, inhalants, sedatives and sleeping pills, hallucinogens, opioids, and ‘other’ drugs—and frequency of use over time (i.e., lifetime use, frequency of recent use (past three months), frequency of a strong desire or urge to use (past three months), frequency of health, social, legal or financial problems related to use (past three months), frequency with which use interferes with role responsibilities (past three months)) and also whether anyone else has ever expressed concern about the participant’s use or whether the participant has ever tried to cut down use of the substance. Risk scores for each substance were categorized as: low, moderate, or high (Cronbach's alpha ranged from a low of 0.74 for hallucinogens to a high of 0.94 for methamphetamine; all values were acceptable).

*Current drug use (drug screen by urinalysis)*. A multi-drug test cup was used to assess for the presence of amphetamine, cocaine, oxycodone, marijuana, phencyclidine, ecstasy, morphine, benzodiazepines, barbiturates, methadone, methamphetamine, and buprenorphine and three adulterants (pH, oxidants, specific gravity), which are qualitatively analyzed to indicate a positive or negative test result [46].

*Depression symptoms* were measured by the Patient Health Questionnaire-8 (PHQ-8). The PHQ-8 is an eight-item measure that that assesses depressive symptoms during the last two weeks using a 4-point Likert-type scale ranging from 0 (not at all) to 4 (nearly every day). A total sum of scores was calculated where higher scores indicate greater depressive symptoms (Cronbach’s alpha = 0.90) [47, 48].

*Post-traumatic stress disorder symptoms* were assessed with the primary care PTSD screen (PC-PTSD), comprised of four items assessed on a yes = 1/no = 0 scale. Items were summed to create a PTSD score. The PC-PTSD score ranges from 0 to 4, with a score of 3 or higher considered the cut point for PTSD screening purposes (Cronbach’s alpha = 0.82) [49].

*Mental health and substance use treatment*. We assessed by self-report engagement in any substance use treatment in the past (e.g., outpatient drug treatment, methadone maintenance treatment program, 12 step or self-help meetings like Alcoholics Anonymous [AA] or Narcotics Anonymous [NA]) and mental health treatment (e.g., inpatient, outpatient, psychotropic medications) (yes/no).

We assessed *anticipated future life outcomes* with the Life Expectancies Scale, a scale that captures a range of future outcomes; namely, whether the participant expects to finish high school, finish college, be employed for long periods as an adult, receive welfare/public assistance for at least a year as an adult, have the career or job they want as an adult, have children, have a long-term love relationship, be comfortable financially, have good family relationships, and live to ages 70, 50, and 30 years, or already has done so. Items were assessed on a 5-point Likert-type scale (not at all, a little, somewhat, very, has already occurred). Items were recoded to indicate whether the item was rated as “very” likely or had already occurred (yes/no). Welfare/public assistance had a positive correlation with the other items and was not reverse-coded. We calculated an overall score as the proportion of items with a score of 1 where higher scores indicate more optimistic anticipated future life outcomes and more outcomes achieved (range 0 to 1, Cronbach’s alpha = 0.75) [36].

### Primary outcomes (HIV care continuum indices)

HIV viral load was assessed via laboratory report and coded on the log_10_ scale [50]. We present the mean and SD and also coded HIV viral load status as suppressed (≤ 200 copies/mL) or non-suppressed (> 200 copies/mL) [51].

We assessed whether participants were well-engaged in HIV care (yes/no) [52, 53]. This variable was comprised of two indices: whether two or more HIV primary care appointments were attended in the past year, an accepted minimum [54], and whether the participant had not missed three or more HIV primary care appointments past year without prior cancellation, as missed visits are independently associated with mortality [55].

### Qualitative interview template

Qualitative interviews were guided by a semi-structured template developed by the research team, which included experts on the HIV care continuum, young and emerging adults, AABL persons living with HIV, sexual and gender minorities, and immigration, and a community advisory board made up of AABL young and emerging adults living with HIV. The template was structured as a series of questions and prompts. The main purposes of the guide were to understand the participant as a whole person, understand factors that promote or impede engagement along the HIV care continuum and their causes and meanings, and explore domains that were not included in the structured assessments but that might be important (e.g., spirituality). The template directed the interviewer from general to more specific questions in each domain. The template is included as supplementary material.

### Quantitative data analysis

We used descriptive statistics to summarize HIV viral suppression and care engagement outcomes, socio-demographic and background characteristics, and self-regulatory resources. We present descriptive data for the cohort as a whole, and for the HIV virally suppressed and non-suppressed subgroups. Because of the large number of variables, and to reduce the changes of erroneous inferences, we do not carry out tests of bivariate statistical significance between the subgroups, but present these data for the purposes of description. To estimate associations between socio-demographic and background factors, self-regulatory resources, and the two outcomes, we used binary logistic regression. For each outcome, variables were entered in two blocks, forming two models for comparison: (1) socio-demographic and background only; and (2) socio-demographic, background, and self-regulatory resources; namely, tobacco, alcohol, cannabis, and other drugs at a moderate- or high-risk level, depression symptoms, the PTSD score, and anticipated future life outcomes. Organizing the variables into blocks allowed us to determine what self-regulatory resources added to background variables. Coefficients estimated by binary logistic regression are log odds ratios, and exponentiating the coefficients leads to odds ratios (ORs), which describe how a one-unit change in the explanatory variable multiplies the outcome variable odds. Associations were reported as ORs with 95% confidence intervals. The R statistical computing program was used for the logistic regression analysis, including tests of significance and confidence intervals [56]. All tests of statistical significance were two-tailed, *p* < 0.05 was considered significant, and p ≤ 0.10 was considered non-ignorable. For the purposes of development of the qualitative research questions, results were considered non-ignorable at the p ≤ 0.15 level.

### Qualitative data analyses

Analyses of qualitative data followed a directed content analysis approach that was both theory-driven and inductive [57]. Analyses were carried out in Dedoose, a cross-platform application for analyzing qualitative and mixed methods research. We started with an initial list of “start codes” and their operational definitions generated by the primary qualitative analyst. This start code list was informed by social action theory. Using this code list, three analysts coded interview transcripts. During the coding process, codes were refined, clarified, and/or broadened. Discrepancies in codes and coding between the data analysts were resolved by consensus. Interview transcripts were recoded using the final coding frame. We formed an interpretive community to organize codes into themes and sub-themes in an iterative process. The interpretive community was led by the three primary analysts and included members of the research team and study investigators. Methodological rigor of the analysis was monitored using an audit trail of process and analytic memos [58]. The primary analysts and the interpretive community attended to the potential effects of the team’s positionality related to power and privilege, sex, gender, race/ethnicity, citizenship status, health, and socioeconomic status throughout the data collection process through reflection and training that focused on how these factors might affect interviewing and data analytic processes [59, 60].

### Developing the qualitative research questions

Quantitative findings were used by the research team to generate a set of research questions that could contextualize and advance understanding of the quantitative findings and that could be addressed using the qualitative data. We considered the quantitative descriptive results and variables in the logistic regression that met or approached statistical significance at a p ≤ 0.15 level (shown in bold in Tables 4 and 5), and considered gaps in the larger literature. Drug use was prevalent at moderate-to-high risk levels (particularly cannabis, tobacco, and alcohol) and appeared higher among those not virally suppressed, and the multivariable analyses suggested the importance of mental health symptoms and marijuana use at a high-risk level as barriers to HIV management. We focused the qualitative analysis mainly on these domains. The specific qualitative research questions were: What are participants’ perspectives on the role substance use plays in their lives and what are its contexts; how does mental health influence substance use and vice versa; and what are the strategies and resources participants used to manage substance use and HIV, including treatment. We also attended to the other self-regulatory resource domains in the model; namely, anticipated future life outcomes and spirituality. Spirituality was not included in the quantitative assessment.

### Data integration procedures

Quantitative and qualitative analyses were conducted and then we used two methods to integrate the quantitative and qualitative results. First, we used the joint display method following procedures described by Fetters and colleagues [61]. A joint display is a visual tool that consist of a side-by-side visual presentation of results. The process of creating the joint display is intended to bring about new insights beyond the information gained from the separate quantitative and qualitative results. Thus, joint displays are both a method and a cognitive framework for data integration and facilitate the production of new inferences [61]. Data integration was carried out by the research team. First, beginning with the major quantitative findings, the research team assessed areas of convergence and divergence between the quantitative results and the primary themes in the qualitative data analysis. To do so, an informational matrix was used to compare results at a granular level (finding-by-finding). The results from this data integration effort were summarized and presented in a joint display table.

Second, we carried out a themes-by-statistics analysis. We separated participants into two categories: those with and without HIV viral suppression at the time of study enrollment. We then compiled the qualitative data statements substance use in a table, and examined if the density and content of statements on substance use differed by viral suppression status, allowing for meta-inferences [62]. The results from these two methods of data integration were then compared to inform interpretation of results.

## Results

### Quantitative results

#### Sociodemographic and background characteristics

As shown in Table 1, participants ranged in age from 19 to 28 years (mean = 25 years; SD = 2 years). The majority were Latine/Hispanic (59%). Almost all (96%) were assigned male sex at birth and the majority (66%) were cisgender. Almost all (93%) identified as gay, lesbian, pansexual, bisexual, or queer. Almost half (49%) were born outside the US or Puerto Rico, and one-third (33%) reported Spanish as their primary language. The mean ACES-R score was 7 (SD = 4). Participants had been diagnosed with HIV for an average of 4 years (SD = 3 years).

**Table 1:** Sociodemographic and background characteristics [mean (SD) or %, (N)]

	**Total (*N* = 271)**	**Suppressed (*N* = 219)**	**Not Suppressed (*N* = 52)**
Current age, in years	25.2 (2.38)	25.2 (2.37)	25.1 (2.46)
Range [Min Max]	[19.0, 28.0]	[19.0, 28.0]	[19.0, 28.0]
*Race/ethnicity*			
Latine or Hispanic	58.7 (159)	59.8 (131)	53.8 (28)
African American/Black or bi- or multi-racial (Non-Hispanic)	41.3 (112)	40.2 (88)	46.2 (24)
*Sex assigned at birth*			
Male	95.6 (259)	95.9 (210)	94.2 (49)
Female	3.3 (9)	2.7 (6)	5.8 (3)
Intersex/other/prefer not to answer	1.1 (3)	1.3 (3)	0 (0)
*Gender identity*			
Cisgender	66.1 (179)	64.8 (142)	71.2 (37)
Transgender, gender expansive, gender non-binary, gender queer or otherwise not cisgender	33.9 (92)	35.2 (77)	28.8 (15)
Lesbian, gay, bisexual, queer, or other non-heterosexual sexual orientation (LBGQ)	93.4 (253)	93.6 (205)	92.3 (48)
Born outside US or Puerto Rico	49.1 (133)	51.1 (112)	40.4 (21)
Engaged in activities in Spanish	32.5 (88)	35.6 (78)	19.2 (10)
Financial hardship—Could not meet needs for necessities in past 6 months (rent, food, utilities), at least once	81.9 (222)	81.3 (178)	84.6 (44)
Adverse Childhood Experiences (ACES-R) score (range 0–14)	7.19 (3.72)	7.28 (3.63)	6.77 (4.12)
Years since HIV diagnosis	3.90 (2.86)	3.81 (2.88)	4.27 (2.80)
Range [Min, Max]	[0, 14.0]	[0, 14.0]	[0, 12.0]

Table 2 shows the lifetime prevalence of substance use, substance use risk scores, and drug screen by urinalysis. Regarding the lifetime prevalence of use, 54% reported lifetime tobacco use, 79% reported lifetime alcohol use, 68% reported lifetime cannabis use, 24% reported lifetime cocaine use, 22% reported lifetime methamphetamine use, 35% reported lifetime inhalant use, and 28% reported lifetime hallucinogen use. Regarding risk scores, 38% of participants reported tobacco use at a moderate-risk level. Alcohol use at a moderate-risk level was reported by 35% of participants. Cannabis use at a moderate-risk level was reported by 51% of participants and at a high-risk level by 3%. The most common drugs found in the urinalysis were marijuana (52%), methamphetamine (13%), amphetamine (13%), and MDMA (10%).

**Table 2:** Substance use (%, N)

	**Overall (*N* = 271)**	**Suppressed (*N* = 219)**	**Not Suppressed (*N* = 52)**
*Lifetime use*			
Tobacco products	53.9 (146)	52.5 (115)	59.6 (31)
Alcohol use	79.3 (215)	79.0 (173)	80.8 (42)
Cannabis	68.3 (185)	65.3 (143)	80.8 (42)
Cocaine	24.4 (66)	22.8 (50)	30.8 (16)
Prescription stimulants	15.5 (42)	15.1 (33)	17.3 (9)
Methamphetamine	21.8 (59)	18.3 (40)	36.5 (19)
Inhalants	35.4 (96)	33.8 (74)	42.3 (22)
Sedatives or sleeping pills	17.3 (47)	15.1 (33)	26.9 (14)
Hallucinogens	28.4 (77)	25.6 (56)	40.4 (21)
Prescription opioids (taken non-medically)	11.1 (30)	10.5 (23)	13.5 (7)
Heroin	0.7 (2)	0.9 (2)	0% (0)
Other drugs	3.3 (9)	1.8 (4)	9.6 (5)
*Injection drug use*			
Ever injected drugs	6.6 (18)	6.8 (15)	5.8 (3)
(If yes) Injected drugs in the past 6 months	55.6 (10)	46.7 (7)	100 (3)
*WHO ASSIST Risk scores*			
Tobacco products			
Lower risk	57.6 (156)	60.3 (132)	46.2 (24)
Moderate risk	38.4 (104)	35.2 (77)	51.9 (27)
High risk	4.1 (11)	4.6 (10)	1.9 (1)
Alcohol			
Lower risk	62.4 (169)	63.0 (138)	59.6 (31)
Moderate risk	34.7 (94)	33.8 (74)	38.5 (20)
High risk	3.0 (8)	3.2 (7)	1.9 (1)
Cannabis			
Lower risk	40.2 (109)	43.8 (96)	25.0 (13)
Moderate risk	51.3 (139)	48.9 (107)	61.5 (32)
High risk	8.5 (23)	7.3 (16)	13.5 (7)
Substances Other Than Tobacco, Alcohol, & Cannabis			
Lower risk	63.1 (171)	65.3 (143)	53.8 (28)
Moderate risk	29.9 (81)	28.3 (62)	36.5 (19)
High risk	7.0 (19)	6.4 (14)	9.6 (5)
*Urinalysis results at baseline (N* = *249)*			
Amphetamines	12.9 (32)	10.9 (22)	20.8 (10)
Benzodiazepines	0.4 (1)	0.5 (1)	0 (0)
Cocaine	2.8 (7)	2.0 (4)	6.3 (3)
Marijuana (THC)	52.2 (130)	47.8 (96)	70.8 (34)
Methamphetamine	12.9 (32)	10.9 (22)	20.8 (10)
Opiates/Morphine	4.8 (12)	5.0 (10)	4.2 (2)
Phencyclidine (PCP)	0.4 (1)	0.5 (1)	0 (0)
MDMA (Ecstasy)	10.4 (26)	8.0 (16)	20.8 (10)

Table 3 displays mental health symptoms and engagement in treatment for substance use or mental health concerns over the lifetime. The average score on the PHQ-8 depression index was 7.3 (SD = 6.1) with a range from 0–24. The average score on the PTSD index was 1.7 (SD = 1.6) with a range from 0–4. Regarding substance use treatment, 12% had engaged in detoxification for alcohol and/or drugs, 10% in 12 step or self-help meetings like Alcoholics Anonymous or Narcotics Anonymous, 7% in outpatient alcohol and/or drug treatment, and 7% in inpatient residential alcohol and/or drug treatment. Regarding mental health treatment, 28% had ever engaged in outpatient mental health treatment, 25% in support groups, 23% had taken medication for mental health issues, and 14% had engaged in inpatient residential inpatient mental health treatment. Rates of service use in the past six months were low for substance use (6% engaged in detox, 3% in outpatient, 4% in Alcoholics Anonymous or Narcotics Anonymous) and mental health (15% outpatient, 11% medication; data not shown on Table 3). Regarding anticipated future life outcomes, 89% expected to graduate high school or had done so, 48% expected to finish college, 64% expected to be employed for long periods as an adult, 49% expected to have a long-term love relationship, 54% expect to have good family relationships, and 55% expect to live to age 70. Although we did not conduct tests of significance, we observed a general pattern of a modestly lower prevalence of positive life expectancies among the subgroup that was not virally suppressed.

**Table 3:** Mental health and treatment use, anticipated future life outcomes (M, SD or %, N)

	**Total (*N* = 271)**	**Suppressed (*N* = 219)**	**Not Suppressed (*N* = 52)**
*Mental health symptoms*			
Depression symptoms (PHQ-8 score)	7.26 (6.14)	7.34 (5.96)	6.92 (6.92)
Median [Q1, Q3]	6.00 [2.00, 11.3]	6.00 [2.00, 11.0]	6.00 [1.00, 11.3]
Range [Min, Max]	[0, 24.0]	[0, 24.0]	[0, 24.0]
PTSD score	1.66 (1.56)	1.67 (1.58)	1.62 (1.50)
Median [Q1, Q3]	1.00 [0, 3.00]	1.00 [0, 3.00]	1.00 [0, 3.00]
Range [Min, Max]	[0, 4.00]	[0, 4.00]	[0, 4.00]
*Substance use treatment—lifetime*			
detoxification for alcohol and/or drugs	11.8 (32)	11.0 (24)	15.4 (8)
inpatient residential alcohol and/or drug treatment	6.6 (18)	6.8 (15)	5.8 (3)
outpatient alcohol and/or drug treatment, such as meeting with a counselor in a clinic	7.0 (19)	7.3 (16)	5.8 (3)
12 step or self-help meetings like AA or NA	10.0 (27)	10.0 (22)	9.6 (5)
methadone maintenance treatment program	0.7 (2)	0.9 (2)	0 (0)
Buprenorphine or Suboxone as prescribed by a health-care provider	1.5 (4)	1.8 (4)	0 (0)
Narcan or Naltrexone as prescribed by a health-care provider	2.6 (7)	2.3 (5)	3.8 (2)
Syringe exchange services	3.0 (8)	1.8 (4)	7.7 (4)
*Mental treatment—lifetime*			
inpatient residential mental health treatment	13.7 (37)	12.8 (28)	17.3 (9)
outpatient mental health treatment	28.0 (76)	29.7 (65)	21.2 (11)
support groups	25.1 (68)	24.7 (54)	26.9 (14)
medication for mental health issues or problem	23.2 (63)	23.3 (51)	23.1 (12)
some other treatment or service for mental health issues or problems	5.5 (15)	5.9 (13)	3.8 (2)
*Anticipated future life outcomes*			
Finish high school	88.9 (241)	89.0 (195)	88.5 (46)
Finish college	48.3 (131)	50.2 (110)	40.4 (21)
As an adult, be employed for long periods	63.5 (172)	63.5 (139)	63.5 (33)
As an adult, be on welfare/public assistance for at least a year	33.2 (90)	31.5 (69)	40.4 (21)
Have the career or job that you want	59.0 (160)	58.9 (129)	59.6 (31)
Have children	32.5 (88)	29.2 (64)	46.2 (24)
Have a long-term love relationship	49.4 (134)	51.6 (113)	40.4 (21)
Be comfortable financially	50.6 (137)	50.2 (110)	51.9 (27)
Have good family relationships	54.2 (147)	55.3 (121)	50.0 (26)
Live to age 70	54.6 (148)	56.2 (123)	48.1 (25)
Live to age 50	68.3 (185)	68.9 (151)	65.4 (34)
Live to age 30	81.9 (222)	82.2 (180)	80.8 (42)

#### Primary outcomes

A total of 72% were well-engaged in HIV care, the primary HIV care outcome. A total of 19% of participants did not evidence HIV viral suppression at enrollment. HIV viral load on a log10 scale ranged from 2.30 to 6.13 copies/mL (data not on Tables).

### Logistic regression models

Table 4 displays the results of the logistic regression relating self-regulatory resource and background variables to HIV care engagement. As a group, the demographic block was associated with HIV care engagement (Χ^2^(df = 9) = 28.3; *p* < 0.001). However, the self-regulatory resources block was not uniquely associated with HIV care engagement after taking demographic variables into account (Χ^2^(df = 11) = 12.9; *p* = 0.297). In the model with only demographic variables, completion of study activities in Spanish increased the odds of being well-engaged in care (OR = 2.66; p = 0.020) and each 1-unit increase in the ACES total score reduced the odds of being well-engaged in care (OR = 0.91; p = 0.017). To add detail for interpretation based on marginal effects (predictions averaging over other variables in the model), for an otherwise typical AABL emerging or young adult, conducting activities in Spanish increased the expected percentage well-engaged in care from 66 to 85%. Likewise, increasing the ACES total score from zero to its maximum (14) reduced the expected percentage well-engaged in care from 84 to 59%. In the model with both demographic and self-regulatory resource variables, while none of the individual variables was statistically significant, both PHQ-8 depression scores (OR = 0.95; *p* = 0.098) and PTSD scores (OR = 0.84; *p* = 0.107) seemed to reduce the odds of being well-engaged in care.

**Table 4:** Multivariable logistic regression models for the HIV care outcome (being well-engaged in HIV care)

	Model 1	Model 2
Age at enrollment	0.948 [0.826, 1.085]	0.965 [0.834, 1.113]
	0.443	0.628
Years living with HIV	0.937 [0.842, 1.042]	0.936 [0.837, 1.048]
	0.231	0.249
Cisgender	0.675 [0.351, 1.267]	0.572 [0.282, 1.125]
	0.228	0.112
LGB sexual orientation (non-heterosexual)	1.479 [0.807, 2.718]	1.466 [0.775, 2.781]
	0.205	0.239
Black or multiracial race/ethnicity (non-Hispanic)	0.882 [0.447, 1.718]	0.821 [0.398, 1.669]
	0.712	0.587
Engaged in activities in Spanish	2.661 [1.182, 6.189] *	1.707 [0.685, 4.352]
	0.020	0.254
Education: Less than high school	0.931 [0.446, 2.012]	0.779 [0.341, 1.830]
	0.852	0.558
Not enough money in past 6 mos for rent, food, or utilities	0.541 [0.224, 1.206]	0.558 [0.221, 1.308]
	0.149	0.195
ACES score (0–14)	0.906 [0.835, 0.981] *	0.959 [0.875, 1.049]
	0.017	0.361
Tobacco at moderate-risk level		0.890 [0.453, 1.755]
		0.735
Tobacco at high-risk level		1.738 [0.350, 10.153]
		0.514
Alcohol at moderate-risk level		1.298 [0.673, 2.553]
		0.442
Alcohol at high-risk level		0.688 [0.109, 4.151]
		0.679
Cannabis at moderate-risk level		0.849 [0.404, 1.766]
		0.663
Cannabis at high-risk level		0.569 [0.180, 1.831]
		0.337
Other drugs at moderate-risk levels		1.021 [0.509, 2.083]
		0.954
Other drugs at high-risk level		0.577 [0.176, 1.852]
		0.354
Depression symptoms (PHQ-8 score)		**0.952 [0.898, 1.009] + **
		**0.098**
PTSD score		**0.836 [0.671, 1.040]**
		**0.107**
Anticipated future life outcomes		0.680 [0.163, 2.783]
		0.592

Estimates are odds ratios with 95% confidence interval; a p-value appears below each interval estimate

Table 5 shows odds ratios relating self-regulatory resources and background variables to HIV viral load suppression. As a group, neither the demographics (Χ^2^(df = 9) = 14.3; *p* = 0.111) nor the self-regulatory (Χ^2^(df = 11) = 10.5; *p* = 0.485) blocks were significantly related to HIV viral suppression. In the model with only demographic variables, while none of the individual variables was statistically significant, completion of study activities in Spanish (OR = 2.35; *p* = 0.061) and having a high school diploma/GED (OR = 1.95; p = 0.082) seemed to be associated with increased odds of HIV viral suppression. In the model with both demographic and self-regulatory resource variables, while none of the individual variables was statistically significant, cannabis use at a high-risk level seemed to be associated with reduced odds of HIV viral suppression (OR = 0.31; p = 0.068).

**Table 5:** Multivariable logistic regression models for HIV viral load suppression

	Model 1	Model 2
Age at enrollment	1.014 [0.872, 1.177]	1.022 [0.873, 1.195]
	0.852	0.780
Years living with HIV	0.955 [0.847, 1.077]	0.948 [0.836, 1.075]
	0.451	0.402
Cisgender	0.662 [0.315, 1.338]	0.627 [0.284, 1.323]
	0.261	0.231
LGB sexual orientation (non-heterosexual)	1.632 [0.838, 3.194]	1.744 [0.870, 3.532]
	0.150	0.118
Black or multiracial race/ethnicity (Non-Hispanic)	1.206 [0.575, 2.502]	1.281 [0.589, 2.763]
	0.616	0.528
Engaged in activities in Spanish	2.354 [0.976, 5.932] +	1.824 [0.673, 5.142]
	0.061	0.243
Education: Less than high school	0.512 [0.243, 1.109] +	0.521 [0.226, 1.230]
	0.082	0.129
Not enough money in the past six months for rent, food, or utilities	0.711 [0.279, 1.646]	0.674 [0.253, 1.634]
	0.446	0.402
ACES score	1.039 [0.953, 1.132]	1.040 [0.945, 1.146]
	0.382	0.426
Tobacco at moderate-risk level		0.575 [0.279, 1.176]
		0.130
Tobacco at high-risk level		3.132 [0.424, 65.829]
		0.332
Alcohol at moderate-risk level		1.044 [0.513, 2.165]
		0.906
Alcohol at high-risk level		1.546 [0.173, 37.786]
		0.733
Cannabis at moderate-risk level		0.670 [0.285, 1.515]
		0.344
Cannabis at high-risk level		**0.313 [0.089, 1.114] + **
		**0.068**
Other drugs at moderate-risk level		0.807 [0.385, 1.720]
		0.573
Other drugs at high-risk level		0.621 [0.166, 2.544]
		0.487
Depression symptoms (PHQ-8 score)		1.027 [0.961, 1.100]
		0.436
PTSD score		0.976 [0.764, 1.250]
		0.846
Anticipated future life outcomes		1.028 [0.220, 4.764]
		0.972

Estimates are odds ratios with 95% confidence interval; a p-value appears below each interval estimate

## Qualitative results

### Overview

We found that participants were highly motivated to maintain good health and adhere to HIV medication as part of that larger health goal, whether they were currently taking HIV medication or not. The majority, but not all, were on HIV medication at the time of the interview, but among these, past periods of lapses or breaks in HIV medication and HIV primary care were common. Consistent with the quantitative results, substance use was prevalent in participants’ lives, including in their social networks and commercial venues where social interactions took place (mainly lesbian, gay, bisexual, transgender, and queer [LGBTQ]-friendly venues). The most common pattern of substance use, from participants’ perspectives, was substance use at non-hazardous levels; for example, on weekends and in social venues. Less common was heavy and potentially hazardous use in the recent period. Those using substances socially or occasionally often described periods of heavy use in the past, particularly at younger ages, and these past experiences provided insights into the dynamics of substance use in this population. The determination of whether substance use was social/non-hazardous or heavy/problematic was drawn from participants’ own implied (e.g., having been in “rehab”) or explicit assessments of the adverse effects of substances on their lives.

**Fig. 2:**
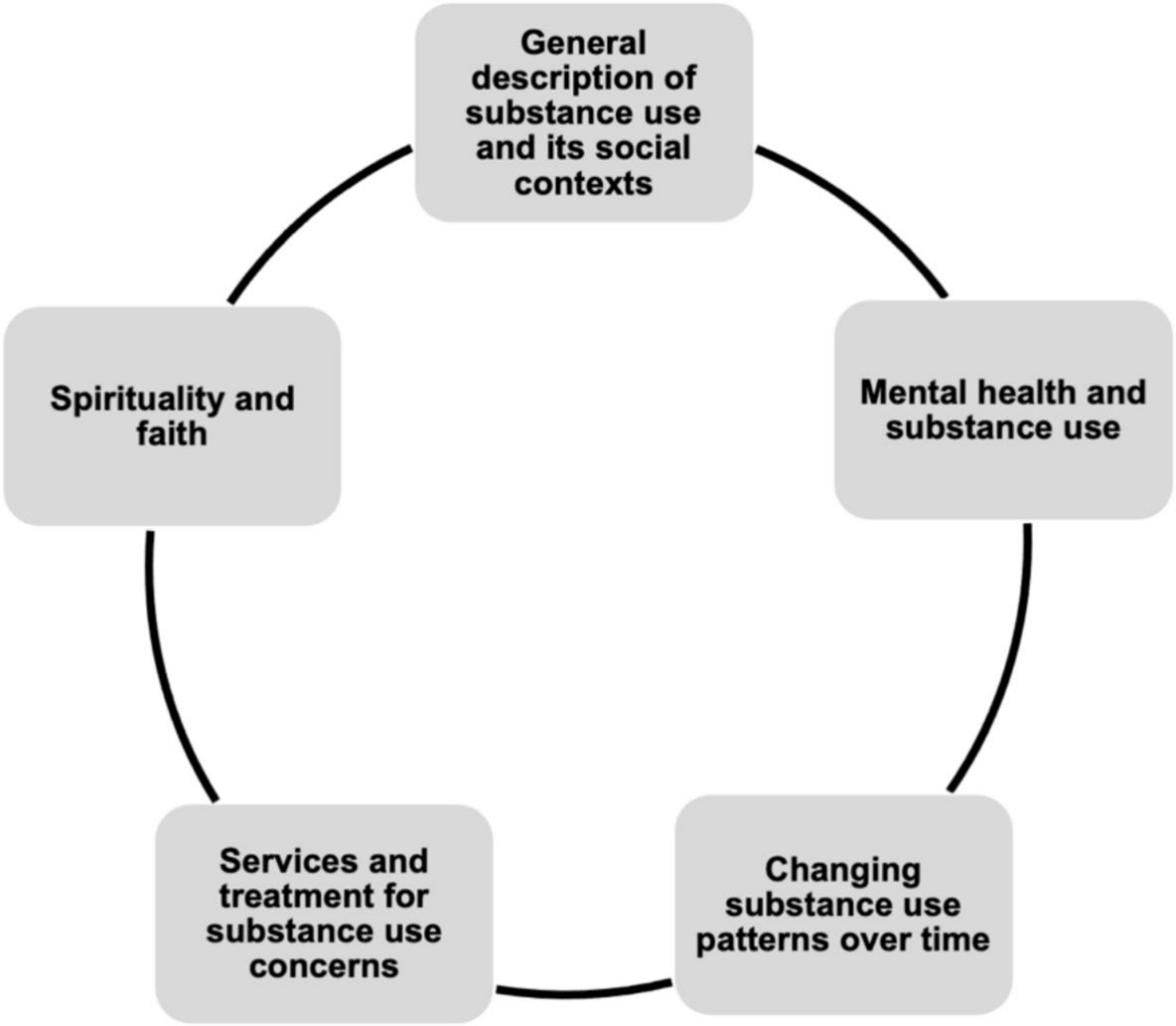
Main themes in the qualitative analysis

Tobacco, marijuana, and alcohol were the most commonly used substances in this analysis, followed by club drugs such as MDMA (Ecstasy) and cocaine. Polysubstance use was also common. Marijuana was not seen as problematic, in contrast to the quantitative findings. Tobacco and nicotine products were described as “addicting” and very difficult to cease. Methamphetamine, while not common, was almost always described as hazardous, and participants feared encountering fentanyl and Rohypnol (“roofies”) in the drug supply or social venues. Substance use was explained as a way to cope with life challenges and manage mental health distress. Substance use did not generally interfere with HIV management. However, in times of heavy use, including polysubstance use or use of methamphetamine, HIV management could be disrupted. Participants might sell HIV medication in times of financial need, exacerbated by substance use. We organized results into the following inter-related themes (Fig. 2.): a general description of substance use and its social contexts; mental health and substance use; changing substance use patterns over time; services and treatment for substance use concerns; and spirituality and faith.

Within these themes were participants’ views on their futures (anticipated future life outcomes), how they hoped to achieve their goals, and factors that promoted or impeded achieving these anticipated future life outcomes. Participants’ anticipated future life outcomes and goals spanned the pragmatic (e.g., getting one’s own apartment) to the ambitious (e.g., starting a business, becoming a therapist, buying a house for their family), and were also typical of the objectives of the general population of young and emerging adults. Participants did not see living with HIV as an impediment to their future life outcomes, although in some cases substance use could be an impediment. In each of these themes we describe strengths and successful HIV or substance use management strategies (e.g., persisting with HIV medication, changing drugs of choice to less hazardous substances), in addition to difficulties and disruptions. Participants are described below using pseudonyms and some identifying details have been changed or removed to protect confidentiality. We use the participant’s preferred pronoun in each description.

### General description of substance use and its social contexts

Substance use was largely a social phenomenon. Marijuana and alcohol were the most common substances described, followed by MDMA (Ecstasy) and cocaine. Substances were used in social venues (generally LGBTQ-friendly venues), including bars and dance clubs, with friends, and on weekends. Participants were introduced to substances by romantic or sexual partners, friends, or in some cases, transactional sex partners in times of economic need. Drugs were discussed on and obtained through dating apps as well. Ivan, a Latine man in his early 20s, described using alcohol and drugs in social settings and noted that his use, while heavy at times, was limited to the weekends:

Well, I smoke a lot of cigarettes, I smoke a lot of weed. I don't drink a lot, like too much. Well, no, yeah, sometimes I drink too much, but it's not too often. OK. I get shit-faced every weekend, but I do drink every weekend. […] And I do cocaine. I like it when I'm drunk, when I'm so drunk or with one of my best friends. We just hang out and it always gets super late. And we'll be watching movies or something, and we're already drunk, so, you know is when the fun comes, but it's not always. […] Just some weed and some coke and some booze every now and then.

Consistent with Ivan’s quote, results highlighted the wide range of drugs available to participants, how substances were embedded in social contexts, and that heavy use might be concentrated on weekends, but not an everyday occurrence. Substance use and polysubstance use were not generally experienced as problematic, as Jordan, a Black man in his mid-20s, noted.

Well, yeah, I smoke. I smoke, I drink. Did I do pills before? Yes. I mean, I’ve tried a lot of drugs. Not trying to sound like a junkie or anything, but I tried a lot of drugs. […] I just didn't do crack. That's a little too hard, but I did a lot. But I mostly stayed with coke, E-pills [Ecstasy] and weed and cigarettes. […] I don't do it, like all the time. I'll do it periodically. Like pills and coke I'll do periodically. Which means, like, I won't do it that much. […] I don't feel like I'm struggling [with drug use] at all. I feel like the only thing I probably am struggling with is cigarettes.

Participants viewed substance use as a prominent aspect of LGBTQ community life and social venues. Some viewed the prevalence of substances as acceptable or even positive, while others, such as Dante, a Latine man in his late 20s, expressed concern, particularly about methamphetamine:

What's upsetting is whoever dropped the bomb, the drug bomb, inside of the LGBTQ community, it has turned them out drastically. From the stories I know it [methamphetamine] used to be a Caucasian [drug]. Now everybody is doing it. […] It seems like everybody in [the local area] gets high. […] Yes. Crystal meth. Now they give a nickname called Tina or T. It seems like everybody in the LGBTQ+ community is strung out and so bad on it. It's scary because these people are a part of my community. […] You just like, oh my God, why?

Some participants did not use substances at all, did not use certain substances, did not use them often, or used them less commonly now than in their younger years. These patterns were related to the desire to maintain health, but not necessarily HIV-related health specifically (“I've always kind of prioritized my health and my well-being. I'm an avid weed smoker, but really that’s the only substance I use”); a wish to avoid substance use problems based on observations of the struggles of family and friends (“that's why I don't use drugs because well I was in an environment of drug addiction and I decided not to have drugs in my life or alcohol or anything”); and lessons learned from past personal substance use challenges. In some cases, participants’ future life goals prompted them to stop or reduce substance use (“I quit smoking weed because I want to be a flight attendant”).

### Mental health and substance use

Struggles with stress and mental health symptoms were pervasive. Depression, anxiety, and attention deficit hyperactivity disorder were mentioned most often. Some participants reported significant trauma in early childhood, trauma related to migrating to the U.S., and trauma in relationships. Further, receiving the initial HIV diagnosis was experienced as distressing or traumatic in many cases as Hugo, a Latine man in his late 20s, described:

It was a bad experience, I would say, because it was confusing. Like, nobody really knew what was going on. […] I was I work the day before, and I remember I had this lump on my neck and my lymph nodes were swollen. […] And when I found out, I cried a lot. And then I was, like, kind of depressed, but, like, I would say about a year. And then my mom let me, you know, go through my feelings.

Substance use was described as a way to cope with the initial HIV diagnosis, ongoing HIV management, life challenges, and mental health symptoms generally. For example, Andres, a Latine man in his late 20s, reported being rejected by his family, in part because the family objected to Andre’s relationship with a partner who the family considered abusive, which, in turn, contributed to feelings of isolation and depression. Andres’s partner introduced him to methamphetamine by injection, and this eventually interfered with Andres’s HIV management. Andres described this period of time as follows:

Honestly, I didn't forget [to take HIV medication]. But I didn't want to take it. […] It was already something that I didn't even take care about anymore, I didn’t care about me, I mean, I didn’t care about how I was dressed and all that. I didn't even have any hygiene. It's something that you forget, […] your private life and everything and that you have a family, you forget everything, you lose track of time. I just wanted to be high. Just wanted to be having sex. […] It was a matter of me feeling worthy, that is, I didn’t think anything was worth it. And I didn't have a part of myself anymore. I didn't have a connection to my life. […] I was already leading a messy life until I finally said"enough, enough."

Compared to other substances, it was difficult for participants to cease using methamphetamine, as Sophia, a 24-year-old mixed-race transgender woman described: “I also have a struggle with [methamphetamine] addiction. It lasted two and a half years. It was crazy.”

Other than marijuana, substances were seen as helpful to reduce stress in the short-term, but were not optimal or effective treatments for mental health concerns in the long-term. Darrell, a Black man in his late 20s, described the period of time after his father died:

Well, after I went down the dark path, I had ended up doing drugs or whatever. I basically really gave up on living life or whatever or whatnot. […] I was doing it [methamphetamine] practically every day compared to now. It's like every blue moon [now]. […] It allowed me to escape reality or whatever. […] Once it was done and over with, I came back to reality like, yeah, I'm still dealing with the depression. […] In the beginning it was a problem [for HIV management] or whatnot. […] Now I can do [drugs] with it [HIV medication].

Thus, as Darrell noted, substance use did not eliminate mental health symptoms, and using methamphetamine heavily could disrupt HIV management, as well as achieving any life goals (“I was looking for an apartment, you know, couldn't get one”).

Generally, marijuana was described as helpful. But, the frequent or heavy use of other substances in the context of mental health symptoms interfered with HIV management, as Trayvon, a mixed-race man in his late 20s, discussed:

[Taking HIV medication] varies on my mood. You know, because sometimes I'm in the mood, sometimes I'm not. […] When I'm not in a good mood, it's just everything kind of crumbles. I deal with depression and all these other mental health, you know, illnesses and things. It just it can really take you by storm and it can be overwhelming. […] Marijuana [helps]. I used to drink a lot. […] I stopped drinking because when I drank, I need something to balance me out. That's when you need some cocaine. […] [Now] I try not to overload myself with too much since I'm, like, extremely emotional. […] But [now] when it comes, I'm ready for it. [I] meditate.

Thus, marijuana was not seen as problematic, but alcohol, cocaine, and methamphetamine could be. Participants often used substances heavily at the time of their HIV diagnosis as a means of coping. Mental health distress and hazardous substance use tended to co-occur and did not necessarily disrupt HIV management, but could in some cases, such as when mental health distress was serious and/or substance use was frequent.

### Changing substance use patterns over time

It was more common for participants to describe past substance use problems than current problems. Participants typically found ways to better manage substance use and HIV over time, often through trial and error and with the support of HIV care settings, family, friends, and community. This was true of mental health distress as well. Thus, periods of heavy substance use were episodic and often related to traumas or losses. Those with periods of heavy use discussed how they transitioned from heavy or problematic substance use to a period of abstinence or casual/social use. They also commonly changed the type of substances they used, such as shifting from alcohol, cocaine, “pills,” or methamphetamine to marijuana, a type of harm reduction approach. The desire for good health, often linked to living with HIV and having access to regular health care, seemed to motivate transitions to less harmful substance use patterns. Some participants described multiple stays in “rehab” but did not specify if these were helpful or not.

For some participants, reducing or changing substance use patterns seemed like a natural change as they evolved over time, as Terrance, a Black man in his late 20s, noted:

That's [marijuana] one thing I don't plan on stopping. I mean, I don't, I don't think so. Maybe in the future it might change. But my mindset on that, I don't think I'm gonna stop smoking marijuana. […] I think that would be the one thing that I am addicted to, and I'm OK with that. I think I'm fine with that. […] In high school, I used to be a pill popper. […] Tylenol 3 [with Codeine] or Percocet. [...] I used to do it. I can't do anymore. I feel like my body can't handle it.

Life circumstances such as family support or stable housing could support a move to non-hazardous substance use but the converse was also true. Participants with little support and serious financial stressors, and in social networks or relationships with other persons using substances heavily, could describe their own use as problematic. Cedric, a Black man in his mid-20s, described himself as frequently “bingeing hard drugs” and, driven by emotional and financial deprivation, being introduced to drug use at the age of 14 by a transactional sex partner (“transactional situations like my sugar daddy. He's the reason why I am on hard drugs. I met him when I was 14. I didn't ask for this problem”). Cedric continued:

[I have been] festering this whole year. Like I just got a job. So it's just like the intermission to the festering session. I kind of don't know how to do anything else other than, like, binge hard drugs. And, like, when I'm not bingeing hard drugs, I'm like, bingeing sleep and mother-son arguments. And hemorrhaging money I don't have. So like I don't really know how to be an adult. I'm not stupid, feel me? […] [But I have an] appetite for drugs. […] I wish I would have not done it [started taking drugs].

Cedric was committed to taking HIV medication and maintaining HIV viral suppression. But, these drug use patterns did interfere with this objective at times. At the time of the interview, Cedric expressed concern about his substance use and desire to stop using drugs, but his narrative reflected the centrality of substance use in his life. He noted:

Like I don't really think about tomorrow. The first thing on my mind usually when I wake up is get a fucking blunt in that mouth. […] You know, marijuana is just like my like, I would like to have a football field or more in between my present consciousness and society. […] And I really just like to bring people joy. Honestly, I don't know how, where or how I got to any of these points. But this is why I got to stop doing drugs.

Cedric had a goal to move from congregate living to an independent housing situation, along with getting back to making art (“art is how I cope”) and starting his new job, suggesting determination and resilience. But, he had engaged in in-patient drug treatment several times, highlighting the severity of his drug use patterns and the fact that substance problems can be chronic and recurring (“like, I went to rehab so many times”).

### Services and treatment for substance use concerns

Health care providers and substance use treatment settings were important resources. Participants commonly found it helpful to involve their health care providers in their concerns about substance use and the potential effects of substance use on HIV medication adherence.

Participants who spoke with their providers about substance reported positive and supportive reactions from providers (“Yeah, he knew [I was using] 'cause I tell him. We're good”). Some participants had been involved with inpatient or outpatient substance use treatment programs, often more than once, and others resided in housing for persons with substance use problems that used either a harm reduction or abstinence-based approach. Results suggested that treatment programs were useful, and played a role in participants bringing substance use to non-hazardous levels, as Hugo, introduced above, described:

I went to rehab for [alcohol] and cocaine. Because I was in the club scene a lot and my friends were party promoters. I feel like maybe I kind of like, try to numb the pain, I would say. […] It [alcohol and drugs] didn't [help] because it made me a different person. And now, like, as I got older, I've learned that I got to see a side of me that I don't like. And now I know that I don't have to do those things to try to, you know, escape a problem or, you know, something in general that happens in my life. No. And yeah, it just wasn't good at the time for me.

These results highlight the complexities of substance use for this population, in light of its availability in the social networks and venues in which participants were embedded, and the perceived utility of substances to address mental health distress and stressors. The HIV care and substance use treatment systems were critical in supporting participants as they worked to manage HIV and achieve their life goals.

### Spirituality and faith

Some participants described their spiritual beliefs as a source of comfort. Participants were commonly involved in organized religion in their early years. But, it was not typical for them to be currently involved with organized religion or attending church, although some did so. For most who discussed religion or spirituality, this distance from organized religion was related to a lack of acceptance of their LGBTQ sexual orientations and/or gender identities on the part of their families (“[My stepfather is] still stuck in the Bible, with what’s written in Leviticus”) and/or the religious institution, or associated with their personal spiritual beliefs. Dylan, a Black genderfluid person in their late 20s, described:

I’m not into religion very much, because it’s very much contradictory. And I’ve had bad experiences with churches and stuff, so, you know. I don’t really do the whole church thing. I believe in God.

For some, belief in God was an important source of comfort. Rafael, a Latine man in his early 20s, described his reliance on God and faith as follows:

I consider that the most important thing for me right now, the most important thing in my life is to always have God's help. I am a person who believes a lot in faith, but if we take it to a material level, I consider that for me right now the most important thing would be to get a stable job. […] I think that also helps me a lot, it's the fact of faith, knowing that I count on God, knowing that God is for me no matter how I lead my life, God is the only person who does not judge me, who does not see me badly, who loves me beyond who I am. And well, I think this is what keeps me standing and what always keeps me firm, and it is the fact of knowing that God is there for me.

Like Rafael, other participants described their faith and spirituality as important resources for them. For some, their faith was a source of hope and strength, as Jalen, a Black man in his late 20s, noted:

It's just like my faith grew over time. […] And it's just like it was this little fire up under me that said, keep going. You know, no matter what they say, keep going, there's hope. There's hope, there's a little bright light on the other side. So, that's what drove me. And that's, you know, that's a daily for me, honestly.

Spirituality was not commonly directly linked to HIV management in these results, and HIV was not a reason most participants did not engage with organized religion. Instead, faith and spirituality supported general wellbeing and resilience, and HIV management was one aspect of the challenges that spirituality could help with.

### Data integration

Table 6 provides a comparison of the quantitative and qualitative results using the joint display framework, and notes if data sources were congruent, discrepant, and if results raise additional research questions. Overall, qualitative results added context to the quantitative results and added meta-inferences. For example, the quantitative data showed that less than a quarter of participants had used methamphetamine, but the qualitative results highlighted that methamphetamine, when used, was generally problematic for participants. In other cases, results were discrepant. For example, quantitative data suggested cannabis at a high-risk level reduced the odds of viral suppression, but the qualitative results highlighted marijuana as helpful to participants and a form of harm reduction when they reduced the use of drugs they considered hazardous and turned to marijuana instead. Another possibly discrepant result had to do with age. The quantitative data did not show age effects (although cross-sectional data are not ideal for capturing change over time), while qualitative findings highlighted that participants commonly improved in their abilities to substance use and mental health over time.

**Table 6:** Joint display to compare, contrast, integrate, and interpret findings

Quantitative finding	Qualitative finding	Comments and Interpretation
More than half (53.9%) had used tobacco products, 38.4% used at a moderate-risk, and 4% at a high-risk level	Tobacco use did not interfere with HIV management or other life goals but was described as very hard to quit. Results suggested that participants wished to stop using tobacco products for health reasons	Qualitative results added context to quantitative results. Interventions to support smoking cessation are needed
Cannabis was used at a moderate-risk level by 51%, and a high-risk level by 9%, 61.5% of those with non-suppressed viral load used cannabis at a moderate-risk level (compared to 49% among those with viral suppression), and cannabis use at a high-risk level seemed to be associated with reduced odds of HIV viral suppression in the multivariable analysis	Cannabis use was common but was not described as a problem and did not appear to interfere with HIV management or other behaviors. Participants often switched from other drugs to cannabis, which can be seen as a form of harm reduction	Quantitative and qualitative results were consistent in that cannabis was common in both sets of results, but discrepant in that the main qualitative results did not suggest cannabis conferred risk to participants. The proportion of those with cannabis use at a high-risk level is small
Twenty-two percent had used methamphetamine in their lifetimes. Methamphetamine use may be higher among those not virally suppressed compared to those suppressed (37% vs. 18%)	Methamphetamine, when reported, generally had serious adverse effects on participants’ lives and was challenging to stop. Formal substance use treatment was often needed to reduce or stop methamphetamine use, often more than once	Qualitative results added context to quantitative results
Lifetime substance use was reported among the participants: 35.4% reported using tobacco in their lifetime, 79.3% reported alcohol use, 68.3% reported cannabis use, 24.4% reported cocaine use, 35.4% reported using inhalants, 21.8% reported using methamphetamine, and 28.4% reported using hallucinogens	Participants shared their experiences of using substances in various social settings, often for recreational purposes. Some participants talked about experiencing heavy substance use at certain points in their lives and later transitioning to abstinence or reducing their substance use as a form of harm reduction	Qualitative results added context to quantitative results
Substance use treatment was less common than mental health treatment but the prevalence of treatment was low overall	Participants discussed substance use treatment more commonly than mental health treatment. Marijuana was described as useful for mental health symptoms. It was not possible to determine if treatment needs were generally met from this data set	Qualitative results added context to quantitative results and results were discrepant to some extent. It is possible substance use treatment is more prominent in participants’ minds than mental health care because substance use problems can have serious adverse effects on their lives and treatment is not sought until problems are serious
Providers’ role in managing substance use was not assessed	Participants who discussed substance use with their healthcare providers reported receiving positive and supportive responses from them	Regarding discussing substance use with providers, this may reflect a strength-based strategy and highlights the critical role that providers have in facilitating engagement and support for individuals navigating their HIV care
The prevalence of positive anticipated future life outcomes was modest overall particularly for social domains (e.g., about half expected to have a long-term love relationship and good family relationships) and longevity. Those with non-suppressed viral load appear to have lower rates of positive outcome expectancies in some domains (e.g., long-term love relationship)	Discussion of anticipated future life outcomes was found in the data but was not common. In general participants appeared optimistic about their futures and were striving to achieve goals. It was not clear from the qualitative data whether or how anticipated future life outcomes related to HIV management, but substance use could interfere with goals	Qualitative data do not contradict quantitative data but research questions remain
PHQ-8 depression index was 7.3 (SD = 6.1) with a range from 0–24 (so rates of depressive symptoms were low overall) and comparable in the suppressed and non-suppressed subgroups	Depression was commonly discussed in relationship to the HIV diagnosis and ongoing HIV management, along with other life events and life stressors. Depression could interfere with HIV management and drive substance use as a way of coping. Participants appeared to find ways to mitigate depression	Qualitative results added context to quantitative results. Quantitative data are current depressive symptoms while qualitative are current and retrospective
The average score on the PTSD index was 1.7 (SD = 1.6) with a range from 0–4 so rates of PTSD symptoms were low overall) and comparable in the suppressed and non-suppressed subgroups	The qualitative data did not specifically identify PTSD but trauma and its various effects were mentioned, and contributed to substance use, which in turn could interfere with HIV management. Participants experienced early childhood and family trauma, trauma migrating to the US, and the HIV diagnosis was often experienced as traumatic or close to it	Qualitative results added context to quantitative results
PHQ-8 depression scores and PTSD scores seemed to reduce the odds of being well-engaged in care	As noted above, mental health symptoms were described as impeding HIV management and results provided insights into how this took place including by triggering substance use. HIV medication was more commonly discussed than HIV care	Qualitative results added context to quantitative results
No age effect in logistic regressions	Qualitative results suggested that participants improve in their abilities to substance use and mental health over time, since the most problematic periods for them were in the past. Participants discussed learning how to manage substance use, mental health, and HIV better over time	Quantitative and qualitative results were discrepant in some respects, although quantitative data are not ideal for capturing change over time. Since mental health disorders and substance use problems can be recurring, preventive interventions are needed
Spirituality was not assessed in the quantitative assessment battery	Spirituality could be an important source of hope. Engagement in organized religion was not common	Qualitative data fill a gap in this paper

Further, our themes-by-statistics analysis examining viral suppression status and substance use added further insights (data not shown on Table 6). More than two-thirds of the participants who had non-suppressed HIV viral loads discussed using substances, including alcohol, methamphetamine, cocaine, and marijuana, among others (68%). Further, these participants commonly had more data related to the theme substances as a coping mechanism. For example, *“Well, after I went down the dark path, I had ended up doing drugs or whatever. I basically really gave up on living life…”* and *“…when I'm smoking or I'm high from the euphoria, I can deal with a lot more situations a little differently or better than I would if I was sober.”* In contrast, among those who evidence suppressed HIV viral load, a smaller number of participants reported currently using substances (26%). Further, among those who reported using who were virally suppressed, use seemed to be reported as more casual or in the past. For example, *“Every once in a while, I might have the urge for, like, a margarita or something, but that's not the normal for me, right? So, I just will take a little sip sip and that's it. So I don't consider myself. So basically, I would consider myself as a non-drinker and non-smoker and non- “druger”, if that makes sense”* and *“No, marijuana is a social thing. I don’t need it.”* This additional mixed methods analysis strengthens our confidence in the marginal quantitative results, which suggest an association between cannabis use at a high-risk level and lower odds of viral suppression (Table 5).

## Discussion

The present study sought to uncover and describe contextual factors in the lives of diverse AABL young and emerging adults living with HIV, focused on self-regulatory resources; namely, substance use, mental health functioning, treatment for substance use and mental health, anticipated future life outcomes, and spirituality. By recruiting participants in the community rather than in medical settings, we captured subpopulations with barriers to research participation and who are typically under-studied in research, such as those with non-suppressed HIV viral load; immigrant, refugee, and asylum-seeking persons; those whose primary language is Spanish; and persons with serious socioeconomic disadvantage. Social action theory provided a useful framework for the present study, as it highlights the importance of “upstream” factors that affect “downstream” health behaviors such as engagement in HIV care and HIV medication adherence, including both risk and protective factors. While some of the domains in the present study, including substance use and mental health, are well-documented in the literature, the present study extends existing research by describing them in detail in this diverse sample, including validated measures and biomarkers, and adding qualitative and mixed methods results for depth and context. Other factors, such as anticipated future life outcomes and spirituality have received less attention in the literature for this population [36, 63]. Thus this mixed methods analysis provides participants’ insights and perspectives on self-regulatory resources and how they operate, both currently and in the past, and each approach (quantitative, qualitative, integrated) uncovered and characterized patterns and relationships among factors not captured by the other.

Substance use was pervasive in participants’ lives, and largely a social phenomenon, similar to their counterpart seronegative LGBTQ peers [64, 65]. Taking together reports of lifetime and recent use and the drug screen by urinalysis, the most frequently used substances were tobacco, alcohol, and marijuana. Stimulants (cocaine, methamphetamine) and “club drugs” such as MDMA were also commonly used, and use of opioids and drug use by injection were not common. Substances were mainly used at low- and moderate-risk levels, but use at high-risk levels, although not the predominant pattern, appeared to be associated with serious adverse consequences on HIV management. The descriptive data further suggest that the prevalence of substance use is higher among those with non-suppressed HIV viral load than suppressed viral load, a finding further supported by the theme-by-statistics analysis. This is consistent with past research that shows that any drug use and polysubstance use are associated with poor HIV viral suppression in this age group [64, 66, 67]. Yet the present study also highlighted the prevalence of non-hazardous substance use in participants’ lives.

The existing research on whether marijuana use is an impediment to HIV care continuum engagement is inconsistent [64, 67, 68]. In the present study, there were discrepancies between the data sources regarding the effects of marijuana use, where quantitative results suggested an association with cannabis at a high-risk level and HIV viral non-suppression, but qualitative results highlighted its importance for coping, mood stabilization, and harm reduction. Paul and colleagues found that among street-involved youth, marijuana was seen as a form of mental health and substance use treatment that was more effective and healthier than the long-term use of pharmaceutical treatments and also safer than alcohol, opioids, and methamphetamine [69]. Thus, consistent with the present study, marijuana was seen as promoting coping and as a form of harm reduction with respect to other drugs. However, Montgomery and colleagues point out that the relationship between marijuana use and HIV continuum of care outcomes is not well-understood in this age group [70]. The present study adds some clarity to the literature in that data suggested marijuana use at a high-risk level was not common, but when present, may reduce the odds of HIV viral suppression, and that marijuana may have utility for harm reduction and as a coping mechanism.

Methamphetamine is a potent stimulant associated with a range of physical and mental health harms, overdose, and mortality, along with the potential for developing methamphetamine use disorders [71, 72]. The present study highlighted challenges some AABL young and emerging adults experience when using methamphetamine, and the strategies they use to reduce harm, including moving to occasional use, switching to other drugs, and seeking formal substance use treatment. Results also highlight the chronic and recurring nature of hazardous methamphetamine use and potential for addiction [73, 74]. Past research indicates that methamphetamine use has serious adverse effects on HIV viral suppression rates [64, 75, 76]. These effects are caused by both behavioral and biological factors (oxidative stress, neuro- and excitotoxicity, and neuroinflammation), since risk for HIV viral non-suppression remains even after accounting for medication adherence and sociodemographic factors [74, 76, 77]. Of concern, there is no FDA-approved medication for methamphetamine use disorder [78]. Cognitive behavioral therapy, behavioral activation, and contingency management treatments show modest efficacy [74]. However, these therapies have limitations and pharmacotherapy for methamphetamine use disorder is needed [78]. Cumming and colleagues identified the most common barriers to treatment access for methamphetamine use disorder [79]. They were all psychosocial in nature and included embarrassment or stigma; belief that treatment was unnecessary; preferring to withdraw alone without assistance; and privacy concerns [79]. In the present study it was not clear whether substance use treatment met participants’ needs, but engagement in such treatment was not common. Further, methamphetamine use may be a cause and a consequence of mental health concerns, complicating treatment [80, 81].

Harm reduction for methamphetamine use and methamphetamine use disorder is a relatively new field. Jones and colleagues point out that understanding motivations for methamphetamine use is important to inform prevention, treatment, and harm reduction strategies [72]. The literature highlights that methamphetamine use is used for pleasure, pain avoidance, increased energy, and enhanced sexual pleasure (called “chemsex) [72]. The present study highlights that methamphetamine is embedded in social networks and LGBTQ venues, and most of those who use methamphetamine found it challenging to stop, although occasional and non-hazardous use of methamphetamine was possible. Results suggest factors driving methamphetamine use in this population include experiences of mental health distress combined with the availability of the drug in social networks. Yet, overall the health, functional, and cognitive outcomes associated with methamphetamine use in younger people, and treatments approaches including harm reduction, are not well understood [82, 83], including among AABL young and emerging adults living with HIV.

Participants in the present study are young and emerging adults, and thus located in a period of the life course characterized by change and transformation and a move toward greater autonomy [16]. In the general population, the central features of these developmental periods are identity explorations, instability, self-focus, feeling “in-between,” and experiencing possibilities [16]. In the general population, the prevalence of drug use for most types of substances is highest in emerging adulthood when compared to other age groups [16]. In part this may be related to substance use as a means of coping with these changes, and because emerging adulthood is a period when optimism is almost universal and expectations for life are high. This, in turn, results in optimism bias, where negative consequences of substance use are not apparent to the emerging adult [16].

However, developmental trajectories vary widely in this period in response to contextual factors, some of which foster positive trajectories and others that impede development [84]. Participants in the present study experience challenges typical of the young and emerging adult periods, along with atypical challenges. However, in contrast to the description of emerging adulthood as a time of optimism, in the present study, anticipated future life outcomes can be described as mixed. For example, only approximately half expect to finish college, have a long-term love relationship, be comfortable financially, have good family relationships, or live to age 70. Certainly, to be effective, interventions must be congruent with the characteristics of the population’s developmental period, and individual differences within the population [81]. These findings suggest the need for interventions tailored to the specific life circumstances of AABL young and emerging adults living with HIV to support optimal HIV, mental health, and substance use behavior. In particular, living with HIV may complicate identity explorations, create instability, and reduce the experience of possibilities. Yet, the majority of participants in the present study evidence HIV viral suppression and substance use at high-risk levels is not common, suggesting substantial resilience and indigenous coping strategies, which can be harnessed for interventions and to support HIV management [85–87].

### Limitations

The study has limitations. First, the self-regulatory resources explored here are distal to HIV care and viral suppression and these factors may interact with others in the social action theory model. Further, we did not seek to make causal inferences in the quantitative data analyses. It was outside the scope of the present study to examine polysubstance use, and we did not determine if participants had substance use disorder diagnoses.

## Implications and conclusion

The present study advances what is known about a set of self-regulatory resources among diverse AABL emerging adults living with HIV. We present the implications of and recommendations drawn from the present study in Table 7. We briefly summarize these implications here. The study highlights the utility of focusing on members of the population with barriers to research and the qualitative and mixed methods approaches. The fact that age- and racial/ethnic disparities in HIV care continuum engagement are serious and persistent indicates the need to invest in research, including intervention studies, to support positive developmental trajectories and wellbeing in this group. Study findings yielded risks that participants face, including with respect to substance use and challenges to HIV management, along with resilience, and also avenues for improved prevention and intervention. In particular, findings suggest the need for frequent screening for substance use and mental health challenges and suboptimal HIV management, and for interventions that take into account variability in developmental trajectories. Further, effective and efficient interventions for tobacco and methamphetamine use appear lacking. Results also suggest that harm reduction is common in this sample and that this promising approach warrants further study.

**Table 7:** Implications and recommendations from the present study

Domain	Implication or recommendation
Community-based studies, qualitative studies	Community-based studies are needed to complement projects carried out in medical settings, since the population of AABL young and emerging adults living with HIV changes and some members of this population have serious barriers to research. Further, qualitative studies are useful for uncovering the population’s views on the causes, meaning, and effect of factors such as those studied here
Coping	Substance use is a common means of coping with stress, life changes, and mental health symptoms in this population. It may be useful to increase the range of coping strategies available to AABL young and emerging adults living with HIV, including psychosocial strategies
Harm reduction	Harm reduction approaches are necessary for AABL young and emerging adults living with HIV and show utility. But, the best ways to apply harm reduction in this population are not well understood and warrant further research
Tobacco use	Tobacco use is common in this population, although smoking is hazardous for persons living with HIV. Smoking is difficult for AABL young and emerging adults living with HIV to stop, highlighting the need for the implementation of interventions and new intervention approaches
Emerging adulthood	Person-context interactions are complex in young and emerging adulthood, and there are many potential pathways to adulthood. Members of this population may reach the markers of adulthood earlier or later their peers in lower-risk contexts and with more resources. It is necessary for services and interventions to take variability in development into account to be effective
Prevention and intervention	Screening is recommended at regular intervals with AABL young and emerging adults living with HIV, focused on social determinants of health, mental health, substance use, and HIV management. Screening results can be used to prevent or treat mental health and substance use disorders and disengagement from the HIV care continuum. At the same time, the fact that age- and racial/ethnic disparities in HIV care continuum engagement are serious and persistent indicates the need to invest in research, including intervention studies, to support positive developmental trajectories and wellbeing in this group

## Supplementary Information

Supplementary Material 1.

## Data Availability

Data are available upon reasonable request from the corresponding author.
