# Supplementary material for: Understanding African American/Black and Latine young and emerging adults living with HIV: a sequential explanatory mixed methods study focused on self-regulatory resources
Source: Int J Equity Health. 2025 May 5;24:120. doi: 10.1186/s12939-025-02492-5 (PMC12051309; doi:10.1186/s12939-025-02492-5)
Supplement: Supplementary file 1 — Supplementary Material 1. [file 12939_2025_2492_MOESM1_ESM.docx]

**SUPPLEMENTAL MATERIAL**

**NYU N4 Connect Qualitative interview template**

**Contact: Marya Gwadz, PhD (mg2890@nyu.edu)**

**Jan 19 2025**

**INTERVIEW SHOULD BE AUDIO-RECORDED IF PARTICIPANT AGREES**

**Main purposes**

- Understand the participant as a whole person
- Understand factors that promote or impede engagement along the HIV care continuum and their causes and meanings
  - What has stayed the same, what has changed, and **why**
- Explore domains that receive little attention in the structured assessment
  - What made you join the study?
  - Developmental challenges (disclosure, relationships, sexual behavior, STIs, partner PrEP, substance use, school/work)
  - Identity
  - Immigration status
  - Harm reduction
  - Treatment norms and counter-narratives
  - Structural and societal factors and influences (CRT)
  - Emotional aversion to HIV care and medication
  - Family acceptance of self as a whole
  - Community resources
  - STI testing
  - IPV
  - Gender affirmation (as appropriate) in social, psychological, medical, and legal domains
- Understand the influences of COVID on participants broadly and with respect to HIV
- Acceptability of EMA (if time allows)

**Sampling**: Participants will be sampled for maximum variability on key indices; namely, change in HIV VS

status [gained or lost VS, sustained VS], years LWH, age, sex, race/ethnicity, sexual/gender minority

status (yes/no), and NYC vs. NJ

**Points to emphasize with participants**

- **The interview is confidential**
- **We will not use your real name or any personal details when we write about the study**
- **We want to understand you as a whole person**
- **No judgment**
- **We want to understand your perspectives on your life, including regarding HIV management**
- **You can decline to answer any question you do not wish to answer and we can take breaks at any time during the interview process**

**BEFORE YOU START THE INTERVIEW – KNOW PARTICIPANT’S VIRAL SUPPRESSION “STATUS” AND USE THIS INTERVIEW TO GET THE PARTICIPANT’S PERSPECTIVE ON THAT STATUS. THIS CAN INCLUDE**

**SUPPRESSED VL AT ENROLLMENT – STAYED SUPPRESSED THROUGH FU1**

**SUPPRESSED VL AT ENROLLMENT – NOT SUPPRESSED AT FU1 OR DURING THE PAST 9 MONTHS**

**NOT SUPPRESSED VL AT ENROLLMENT – SUPPRESSED AT FU1**

**NOT SUPPRESSED VL AT ENROLLMENT - NOT SUPPRESSED AT FU1**

**AND THE SAME IDEAS AT FU2…. WHY DO PEOPLE TAKE, STOP, AND START ART? WHAT ARE THEIR PERSPECTIVES ON THAT INCLUDING CONTEXTUAL AND SOCIAL FACTORS.**

**BELOW YOU WILL SEE YOU WILL ASK THE PARTICIPANT ABOUT THEIR ‘STATUS,’ NOT NECESSARILY TELL THEM WHAT THEIR ‘STATUS’ IS FROM THE STUDY’S PERSPECTIVE. THE PARTICIPANT’S REPORT MAY BE DIFFERENT FROM STUDY RECORDS. THAT IS OK.**

| **Domain** | **Main question, probes and prompts** | **Notes** |
| --- | --- | --- |
| Why joined | Think about when you first joined the N4 study. What prompted you to enroll in N4? |  |
|  |  |  |
| Getting to know the participant | Tell me a little about yourself.  PROMPTS  What do you like to do with your time  What is important to you (music, hobbies, etc)  Who are the important people in your life  What are some recent achievements (work, school, etc)  What are some recent challenges  What are your hopes for the near future  What’s one thing that we really need to know to understand you?  NOTE: Participants may be recent immigrants. This can be explored in this section or later. How does being an immigrant affect perspectives, access, decisions, etc? |  |
|  |  |  |
| Understand factors that promote or impede engagement along the HIV care continuum and their causes and meanings | Since you joined the N4 Connect project, has anything changed in your life?  Including with respect to HIV Care and HIV medication  PROMPTS  Engagement in HIV care   - What do you think led to the change in your ‘engagement in HIV care’? - Can you say more about that? Does anything else come to mind? - How often does your level of engagement change? What are the reasons it changes?   HIV medication taking   - What do you think led to the change in your ‘HIV medication taking’? - Can you say more about that? Does anything else come to mind? - How often does your level of adherence change? What are the reasons it changes?   School  Work  Relationships  Identity (e.g., coming out re: sexual orientation or gender identity changes)  Family  Substance use  Mental Health  Other? |  |
|  |  |  |
| Developmental challenges (STIs) | People between the ages of 16 and 28, who are the focus of N4 Connect, have a lot of new issues to deal with as they get older.  We want to understand your perspectives on how you manage these issues.  We want to talk about **sexually transmitted infections**.  Rates of infections like syphilis, chlamydia, and gonorrhea are high these days.  Are you aware of that?  Are people talking about that?  Is this something you consider in your sexual encounters? How do you manage that? |  |
|  |  |  |
| Had STI (other than HIV) | Are sexually transmitted infections something you worry about or have had to deal with?  Do you consider STIs a minor issue or serious issue?  Can you give me some recent examples?  How did you find out?  Did you get treated? Tell me about that experience. Was it easy? Challenging? |  |
|  |  |  |
| STI testing | Is testing for sexually transmitted infections a regular part of your health care? DESCRIBE  If you could test yourself at home for STIs, would you want to do that? |  |
|  |  |  |
| STIs and safer sex | Having safer sex is a challenge for people, including people living with HIV  How do you define “safer sex?”  When is it easy to have safer sex?   - Type of partner (serious, casual, one-time) - Age of partner - Race/ethnicity of partner - Presumed HIV status of partner - Known HIV status of partner - Location where sex takes place (home, local neighborhood, public space, other) - Drug use (type of drug, quantity, etc) - Other   When is it harder?   - Type of partner (serious, casual, one-time) - Age of partner - Race/ethnicity of partner - Presumed HIV status of partner - Known HIV status of partner - Location where sex takes place (home, local neighborhood, public space, other) - Drug use (type of drug, quantity, etc) - Other |  |
|  |  |  |
| Stigma, feelings | Is it easy or challenging to talk about STIs with partners?  With providers? |  |
|  |  |  |
| Why – what can be done | Why do you think rates of HIV are going up among young adults in New York City, including people living with HIV?  What can health care providers do to help younger people manage their sexual health (PROMPTS CAN INCLUDE MORE EDUCATION, REDUCE STIGMA, MAKE SERVICES EASY) |  |
|  |  |  |
| Substance use | Just a reminder that everything you tell us is confidential.  Many people your age use alcohol and drugs to some extent.  We want to understand the role of alcohol and drugs in your life, if you use alcohol and drugs.   - What substances do you typically use? - With whom do you use? - How often do you use? - What do you like about that substance? (and/or - How does that substance impact/help you?) - Do you have any concerns about your substance use? DESCRIBE |  |
|  |  |  |
| Substance use HIV management | Does the use of alcohol or drugs have any effect on your ability to engage in HIV care or take HIV medications – whether positive or negative? DESCRIBE |  |
|  |  |  |
| Harm reduction | Have you heard the term “harm reduction?”  Harm reduction is a proactive and evidence-based approach to reduce the negative personal and public health impacts of behavior associated with alcohol and other substance use at both the individual and community levels.  An example could be using when other people are present, to reduce the risk of overdose.  Has anyone talked to you about harm reduction? DESCRIBE  What does harm reduction mean to you? How do you define it?  Do you use harm reduction strategies in your own life? Which ones? Do they help? DESCRIBE |  |
|  |  |  |
| HIV care | Note whether attends care in an adult or pediatric setting  Have you been attending HIV care appointments? Why or why not?  **What helps?** What gets in the way?  Can you think of any thoughts that get in the way? How about emotions?  Do you feel like you are avoiding HIV care? EXPLAIN |  |
|  |  |  |
| HIV care 2 | EXPLORE the transition to adult care settings as appropriate  What factors help/hinder going to HIV care? PROBES/EXPLORE   - Substance use - Gender affirmation - Partner violence - Busy with school/work - Racial/ethnic discrimination in the community - Other discrimination |  |
|  |  |  |
| HIV care 3 | (ASK IF NEEDED) Some people have felt unwelcome in HIV clinic settings, for example, when they were not taking HIV medications, or when their viral load levels were detectable. Have you ever felt that way? (DESCRIBE, EXPLORE)  To what extent have you felt welcome and wanted at your HIV health care setting when you have been taking HIV medication with high levels of adherence? (EXPLORE)    What about when you have not been taking HIV medications? (EXPLORE)    EXPLORE whether intersectional identity or aspects of identity affect care engagement |  |
|  |  |  |
| HIV medication – **why DO you take it,** or not | We want to understand WHY some people take HIV medication consistently (if and when they do that) and if they do, **HOW they are able to do that (looking at all sorts of factors)**. It can be hard to explain things like this sometimes, but we will ask you to try.  Do you take HIV medication consistently? Why or why not?  What helps you take HIV medication “as prescribed” or close to “as prescribed?” What gets in the way?    Can you think of any thoughts about HIV medication that help you take medication consistently or that get in the way?  How about emotions that help or get in the way?  Social relationships that help or get in the way? |  |
|  |  |  |
| Younger people – why DO they take HIV medication | Do you think that younger people (under age 30) are more or less likely to take HIV medication consistently than older people?  Why or why not? |  |
|  |  |  |
| VS | Is having undetectable HIV viral load important to you? Why or why not? |  |
|  |  |  |
| Stigma if not taking medication | How do you feel when you are not taking HIV medication?  Do you experience disapproval from others if you are not taking it?  Are you comfortable telling people you are not taking it? |  |
|  |  |  |
| **** REFLECTIONS ON VL “STATUS”** | We want to understand any changes in your medication taking patterns during the study. We want to know what you think about that.  When you started the study, were you taking HIV medication to the point of having undetectable viral load?  Did that change since you started the study? DESCRIBE  ASK PARTICIPANT TO RELFECT ON FACTORS THAT LED TO STABILITY IN STATUS OR CHANGE IN STATUS |  |
|  |  |  |
| Barriers/facilitators | Let’s brainstorm. Is there anything that could change in your life that would make it less likely you’d take HIV medication and reach an undetectable viral load? DISCUSS  Anything that would make it more likely? Why or why not? DISCUSS  PROBES CAN INCLUDE   - Better quality housing - Substance use under better control - Access to harm reduction services - More attention to substance use in study activities - More TANF/entitlements/cash benefits (to make life easier and reduce diversion) - Better relationships with health care providers 0 DESCRIBE - Supportive romantic partner(s) - Supportive family members - Less internalized stigma - More disclosure to others about HIV status - More trust in medications - More doctors and research staff from the same racial or ethnic group as you - Better physical or emotional health - Financial compensation for achieving undetectable viral load - OTHER? |  |
|  |  |  |
| Mental health | How would you describe your mental health?  Has your mental health changed over the past 3-5 years? If so, how?  What do you do to have the best mental health you can have?  Do you experience challenges to good mental health? DESCRIBE  What helps you cope with mental health challenges?  Do you have access to mental health support? What types are most helpful to you? |  |
|  |  |  |
| Stigma ad indigenous coping strategies | HIV stigma refers to irrational or negative attitudes, behaviors, and judgments towards people living with or at risk of HIV.  There are other forms of stigma too, such as stigma directed at people who use drugs.  We have been talking about how you might experience stigma in some of these questions.  Now I want to ask you about how you manage stigma.  First, do you ever experience different types of stigma at the same time? What is that like?  How do you avoid it?  How do you cope with it?  What would help you deal with it better? |  |
|  |  |  |
| N4 | Is there anything you like about the N4 Connect project?   - Computer Assessments - Drug test - Cell phone short interviews - Contact - Resources provided   What do you not like about the study and what can we do better? |  |
| IF TIME ALLOWS |  |  |
| First ART (RAPID ART INTITIATION) | We are interested in how soon after diagnosis people are offered HIV medication  In what year were you first diagnosed with HIV?  How soon after your diagnosis were you offered HIV medication?  (IF RAPID ART WAS OFFERED)  What did the health care provider say?  What did you think and feel about being offered medication right after diagnosis?  What did you do? Did you start medication right away? Why or why not? |  |
| IPV | Some people have told us that violence is an issue in their close romantic and sexual relationships -whether they are violent towards people or people are violent towards them, or both  Is interpersonal partner violence an issue in your life? DESCRIBE  IF YES – How does it affect you?   - Does it affect your ability to manage your HIV? (go to care, take medication). How?   Would you like any kind of help or assistance to manage this aspect of your life that you are not getting? |  |
| Mpox (MONKEYPOX) | New York City has recently been experiencing a rising number of Mpox (formerly called Monkeypox) cases. What have you heard about this?  Have you spoken with friends, family, coworkers, etc. about this? What are those conversations like?  On a scale from 1 to 10, how concerned would you say that you are about Mpox? (EXPLORE)  Have you considered vaccinating yourself against Mpox? Why/not?  Have you experienced any barriers to receiving a vaccine?  Has the recent rise in cases of Mpox changed your activities in any way? (EXPLORE)  Do you see any relation between HIV and Mpox? |  |
| Identity | We want to know how you see yourself at this point in your life and how different aspects of who you are fit together (or not). These questions can be a little challenging to answer but we want to understand how you see yourself.  Do you feel you belong to one or more specific racial or ethnic groups **racial or ethnic group**? DESCRIBE. ASK, What does that mean to you? How important is that to you?  Do you feel you belong to a specific **religious or spiritual group**? DESCRIBE. ASK, What does that mean to you? How important is that to you?  Do you feel you belong to the **LGBTQ community**? DESCRIBE, ASK WHY OR WHY NOT. EXPLORE SEXUAL ORIENTATION AND GENDER IDENTITY SEPARATELY IF APPROPRIATE, ASK, What does that mean to you? How important is that to you?  Does your **age** play a role in how you see yourself (e.g., identify as a young adult or as an adult). DESCRIBE What does that mean to you? How important is that to you?  Does your **sexual orientation** play a role in how you see yourself? DESCRIBE. ASK, What does that mean to you? How important is that to you?  Does your **gender/gender identity** play a role in how you see yourself? DESCRIBE. ASK, What does that mean to you? How important is that to you?  Do you feel you belong to a certain **profession**, or a school community? DESCRIBE. ASK, What does that mean to you? How important is that to you?  Does your **HIV status** play a role in how you see yourself? DESCRIBE.  Does **taking HIV medication** play a role in how you see yourself? DESCRIBE.  Does having **detectable or undetectable HIV viral load** play a role in how you see yourself? DESCRIBE.  Do you feel you belong to the **HIV community**? DESCRIBE. ASK, What does that mean to you? How important is that to you?  What else should we know about how you see yourself or who you are? DESCRIBE  Which of these identities do you experience as most **central to who you are as a person**? Can you talk a bit more about that? | Flensberg Identity Status Interview, pg 20 |
| Identity 2 | Looking back over the past 3-5 years or so, how have you changed?  Positive changes?  Negative changes? | Flensberg Identity Status Interview, pg 20 |
| Identity 3 | You have reflected on the meaning of different groups you may belong to or ways you see yourself including: NAME CATEGORIES FROM LAST QUESTION (IDENTITY 2)  Which of these are most important to you?  How do these identities tie in with one another?  Are there ways that they compete with each other or ways that they are in harmony?  NOTE: These questions are abstract… Might be challenging to answer. | Flensberg Identity Status Interview, pg 20 |
| Identity HIV | How has living with HIV shaped or changed how you see yourself or feel about yourself? DESCRIBE  Has it changed how you see yourself in positive ways?  How about negative ways?  POTENTIAL PROMPTS   - Can you say more about that? - Can you give me an example? |  |
| Developmental challenges (disclosure, relationships, sexual behavior~~, STIs,~~ school/work) | People between the ages of 16 and 28, who are the focus of N4 Connect, have a lot of new issues to deal with as they get older.  We want to understand your perspectives on how you manage these issues.  How do you manage **disclosure of your HIV status?** Is this easy or difficult for you?  How do you manage **sexual orientation, and/or gender identity**? Is this easy or difficult for you?  What about **sexual and romantic relationships**? Are these easy or difficult for you?  What about **friendship**s? Are these easy or difficult for you?  Do you feel connected to a community, or communities? Which ones? What do they mean to you?  What about **school and work**? Is this easy or difficult for you?  What other challenges are you dealing with? |  |
| Family acceptance/family rejection | Tell us about your relationship with your family  In what ways do they understand and support you?  In what ways do they not understand and support you?  (If a member of the LGBTQ+ community) Do they support your LGBTQ/queer identity? |  |
| What could help with developmental challenges | What kind of help do you get managing issues like identity disclosure, status disclosure, relationships, sexually transmitted infections, and school and work?  What kind of help would you like that you don’t have? |  |
| Future self | Think about the next 5 years or so.  Who do you want to be and what do you want to be doing in 5 years?  What would help you achieve this vision? |  |
|  |  |  |
|  |  |  |
| **LAST Q Anything else** | **What else should we know about you and your life, to understand who you are?** |  |
|  |  |  |
|  | END OF INTERVIEW |  |
